# From the Oral Cavity to the Spine: Prevalence of Oral Cavity Infections in Patients with Pyogenic Spinal Infection

**DOI:** 10.3390/jcm13041040

**Published:** 2024-02-11

**Authors:** Fatma Kilinc, Florian Gessler, Johanna Kessel, Daniel Dubinski, Sae-Yeon Won, Anna Tanneberger, Shahram Ghanaati, Vincent Prinz, Marcus Czabanka, Matthias Setzer, Thomas Freiman, Bedjan Behmanesh

**Affiliations:** 1 Department of Neurosurgery, Goethe University Hospital, 60528 Frankfurt, Germany; fatmakilinc@hotmail.de (F.K.); vincentprinz@gmx.de (V.P.); czabanka@med.uni-frankfurt.de (M.C.); setzer@med.uni-frankfurt.de (M.S.); 2 Department of Neurosurgery, University Medicine of Rostock, 18057 Rostock, Germany; florian.gessler@med.uni-rostock.de (F.G.); daniel.dubinski@med.uni-rostock.de (D.D.); sae.yeon@med.uni-rostock.de (S.-Y.W.); thomas.freiman@med.uni-rostock.de (T.F.); 3 Department of Medicine, Infectious Diseases Unit, Goethe University Hospital, 60596 Frankfurt, Germany; johanna.kessel@kgu.de; 4 Department of Maxillofacial Surgery, Goethe University Hospital, 60596 Frankfurt, Germany; anna.tanneberger@kgu.de (A.T.); sharam.ghanaati@kgu.de (S.G.)

**Keywords:** spinal infection, oral cavity infection, OPG, outcome

## Abstract

Background incidence of pyogenic spinal infections has increased in recent years. In addition to treating the spinal infection, optimal care also includes identifying the source of the pyogenic spinal infection and the presence of other infections. The aim of this study is to elucidate the prevalence of oral cavity infection (OCI) within this patient cohort. Methods As part of a prospective study conducted from 2016 to 2021, the number of patients with dental infections was investigated by means of an orthopantomogram (OPG) and subsequent dental examination. Results The presence of an oral infection was investigated in 55 (47%) of 118 patients by an OPG, 29 (53%) of whom had a corresponding abnormality of the oral cavity. In addition to the spinal infection, patients with an oral cavity infection revealed an increased incidence of endocarditis, sepsis and brain abscess. A spinal epidural abscess, a multilevel affection of the infection, and an elevated CRP value were also found in patients with a co-existing oral cavity infection. Back pain assessed at admission and 3 months after surgery was also more pronounced in patients with an oral cavity infection. Neurological deficits were often present in patients with spinal and oral cavity infection. Conclusions The presence of an oral cavity infection has proven to be one of the important factors in the detection of the source of the pyogenic spinal infection. In addition, a pronounced spinal affection and frequent co-infections were seen in patients with an oral cavity infection.

## 1. Introduction

The incidence of spondylodiscitis in western countries has risen over the past few decades. It is a rare disease with high morbidity and long-term sequelae. Unfortunately, it is often recognized and treated late. The main reasons are the demographic change, extended life expectancy and improved access to medical services [1,2,3,4]. The primary site of infection is the avascular intervertebral disc, and the infection can then spread progressively through the vertebral body and into the subligamentous paravertebral area, epidural space and adjacent vertebral bodies. Diagnosis can be confusing because spondylodiscitis may rarely affect only one vertebral body, with or without disc involvement. Such a diagnosis can significantly impact quality of life. In addition to the spinal focus, infections of other organ systems, such as the heart, brain infections and other soft tissues, are reported more frequently. Therefore, the theory of a systemic infection takes on a special significance, as the hematogenous spread as the source of the infection is of particular importance [5,6,7]. Thus, the detection of the source of infection as the cause of hematogenous dissemination is of particular interest. Unfortunately, in almost 50% of the cases, the primary source of infection is not identifiable despite an intense diagnostic work-up, which makes a targeted antimicrobial therapy in particular more difficult [8]. At the same time, determining the cause of the spinal infection is an important goal of a specific treatment. Increasing evidence indicates that oral cavity infections participate in various systemic diseases, such as cardiovascular, pulmonary or metabolic disease. Dental caries is etiologically associated with a number of pathological conditions in some organs, particularly the heart. Endocarditis is often present in spondylodiscitis. Oral infection, spondylodiscitis and endocarditis are closely related. Local oral bacterial infection is also associated with an increase in systemic inflammatory activity [9,10,11,12]. Infections of the oral cavity are most commonly odontogenic in origin and include dental caries, periapical infections, gingivitis, periodontal infections and abscesses, as well as deep fascial space infections [13]. Until now, little is known about the relationship between oral and spinal infections. For this reason, the present study was a prospective investigation of the prevalence of oral infections in patients with a spinal infection.

## 2. Material and Methods

All patients admitted to the authors’ institution with newly developed primary pyogenic spondylodiscitis were prospectively entered into an institutional database. An analysis was performed for all cases treated for spinal infection between 2016 and 2021 at the neurosurgical department of Goethe-University Frankfurt. According to our treatment algorithm, all patients underwent Gd-enhanced MRI and CT scans to confirm the diagnosis of pyogenic spondylodiscitis, extent of infection and bony destruction. A calculated antibiotic regimen was routinely applied as intravenous treatment for six weeks, followed by six weeks of oral antibiotics. If bacteria were identified, empirical broad-spectrum antibiotic therapy was changed to targeted culture-specific sensitive antibiotic therapy. Consultation with an infectious disease team was performed prior to antimicrobial therapy. The treatment was discontinued when CRP and WBC normalized, and clinical as well as radiological evaluation showed improvement. Post-operative spinal infections were excluded from our study. Infections of the cervicothoracic junction (C7/Th1) were counted as cervical spondylodiscitis, and the thoracolumbar junction (Th12/L1) accounted for thoracic and the lumbosacral junction for lumbar cases. On the basis of the result of the OPG, the patients were divided into two groups, one with an infection of the oral cavity and the other without an infection of the oral cavity.

Information, including patient characteristics, radiological features, diabetes mellitus, body mass index (BMI), initial symptoms and 3 months after treatment/surgery, the localization of the infection, number of affected spinal levels, American society of anesthesiologists physical score (ASA), length of hospital stay, functional neurological status at admission and during the course of treatment, were recorded and further analyzed. The neurological status pre- and postoperatively, at discharge, 3 months postoperatively were assessed using the American Spinal Injury Association-Classification (ASIA score) describing patient’s ability and level of neurological independency. Obesity was defined as a body mass index (BMI) > 30 kg/m^2^. Patient comorbidities were assessed according to the Charlson comorbidity index (CCI) [14].

Microbiological diagnostic work-up included two sets of blood cultures (BD Bactec Plus aerobic and anaerobic medium) drawn before the start of antibiotic therapy and in the further clinical course. In addition, intraoperatively swabs were preserved for microbiological analysis. A CT guided biopsy was performed, if possible, in patients after approval of conservative therapy. Coexisting infections, such as endocarditis and cerebral infections were proved undergoing transesophageal echocardiography and cerebral enhanced MRIs. Back pain was assessed using the visual analog scale for pain (VAS) at admission, postoperatively and 3 months thereafter.

Routine laboratory analysis included C-reactive protein (CRP), white blood cells (WBC), hemoglobin, thrombocyte count, international normalized ratio (INR) and serum creatinine level. If the patient had another underlying conditions that required regular monitoring of laboratory values, such as transaminases, these were also analyzed.

In collaboration as part of a project with physicians of the departments infectious diseases, radiology and oral and maxillofacial surgery, we initiated the present study to determine the prevalence of oral cavity infection in patients suffering from pyogenic spinal infection. In addition to identifying the oral infection, an MRI of the head, transesophageal echocardiography and other investigations, such as abdominal sonography, were ordered within the first week after surgery to identify the cause of the spinal infection. Furthermore, the influence of a present oral cavity infection should be investigated on the development and severity of the spinal infection. The purpose of the study was the radiological examination with an OPG and subsequent clinical assessment by oral and maxillofacial surgeons.

Due to the logistical distance to radiology for OPG and the ability to stand for radiology, only patients who were mobile and able to stand could be enrolled and assessed. In some cases, patients who were unable to stand and had significant neurological impairment could still be included and analyzed in this study because cCT was available. This imaging could be used to assess oral infections.

### 2.1. Statistical Analysis

All statistics were performed using SPSS (version 21, IBM, Armonk, NY, USA). Significant values were considered to be *p* < 0.05. Nonparametric tests, included the Mann–Whitney U and Kruskal–Wallis tests, were used to compare groups of data that did not follow a normal distribution. For statistic analysis, a *t*-test was also used. Comparisons of important baseline characteristics and clinical parameters between patients with and without oral cavity infection were made using Fisher’s exact test for categorical variables. Sample size determination and power analysis was performed by using the G-Power software according to Faul et al. [15,16].

### 2.2. Data Availability

The datasets generated during and/or analyzed during the current study are available from the corresponding author on reasonable request.

## 3. Results

To detect the cause of the spinal infection, an OPG was performed in 55 of 118 patients, of whom 29 (53%) revealed an oral cavity infection as the most likely identifiable source of the spinal infection. The comparison of age, gender, localization of spinal infection, presence of obesity, human immunodeficiency virus (HIV) and intravenous drug use (IDU) showed no significant differences between patients with and without an oral cavity infection. The comorbidities recorded by the CCI and ASA were almost identical in both groups. With the exception of CRP, the laboratory parameters obtained did not show any particular abnormalities. CRP was elevated in patients with oral cavity infections, but without reaching a level of significance. Also, without significance, patients with oral cavity infections revealed more spinal epidural abscesses, diabetes, sepsis, brain abscess, bacteremia proven by blood culture and longer hospital stays compared with patients without oral cavity infections, Table 1.

In this study, bony destruction was significantly more frequent in patients without an oral cavity infection, *p* < 0.02; on the other hand, endocarditis in patients with OCI was more frequently detected, *p* = 0.02. Furthermore, patients with OCI showed multilevel affection of the spinal infection in contrast to those without OCI, 34% vs. 5%, *p* = 0.02.

### 3.1. Pain and Neurological Status

In this study, patients diagnosed with OCI reported more and more severe pain on VAS 9 vs. 8, *p* = 0.02, at initial presentation compared to patients without OCI. In addition, initial neurological status on admission was significantly better in patients without OCI, as well as ASIA E 91% vs. 56%, *p* = 0.002.

### 3.2. Specification of the Oral Cavity Infection

**Figure 1:**
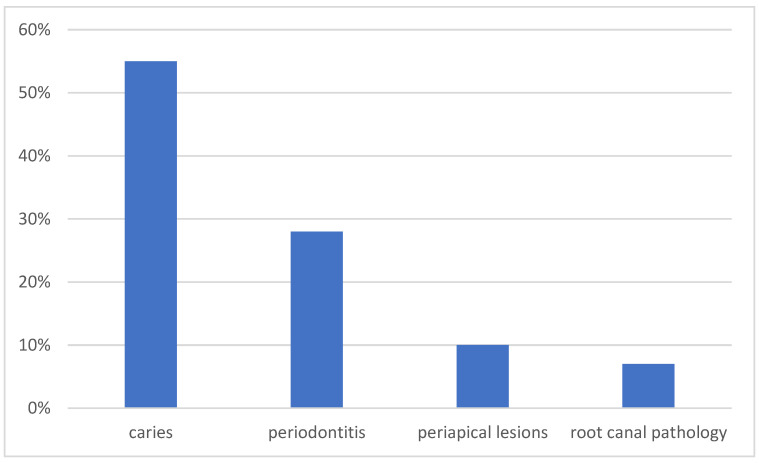
Distribution of the cause of oral infection.

Based on the radiological findings followed by clinical evaluation, in the group of patients with oral cavity infection, caries was diagnosed in 16 patients, peridontitis in 8 patients, periapical abnormalities in 3 patients and dental root infection in 2 patients (Figure 1). Tooth extraction was performed in 12 (44%) cases, with tooth-preserving therapy recommended in 15 (56%) patients (Table 1).

### 3.3. Microbiological Assessment

**Figure 2:**
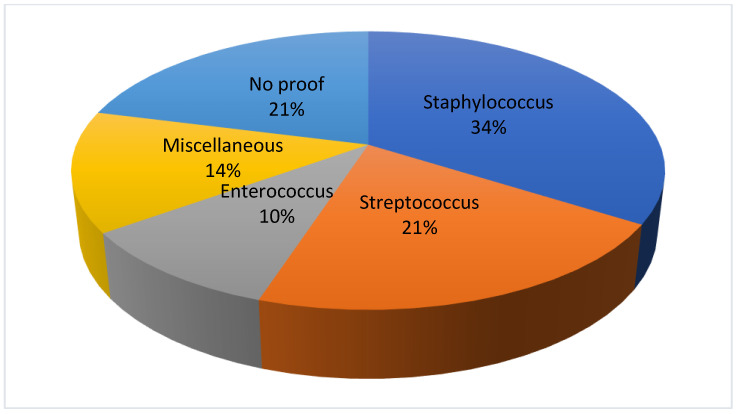
Detection of bacteria in the oral co-infection group.

The pathogen causing the infection was identified by blood culture and intraoperatively obtained swabs from the disc in 23 (79%) patients in the oral coinfection group and revealed a spinal infection caused by staphylococcus in 10 (34%), streptococcus in 6 (21%), and enterococcus in 4 (14%) cases, respectively. In 6 (21%) cases, the pathogen could not be detected (Figure 2).

**Figure 3:**
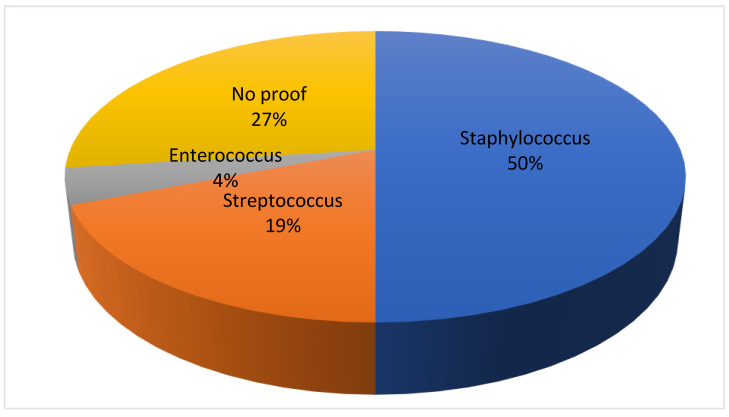
Detection of bacteria in the group without oral cavity infection.

In patients without OCI, a pathogen could be determined in 19 (73%) patients. The spinal infection was caused by staphylococcus in 13 (50%) patients, followed by streptococcus in 5 (19%) cases and by enterococcus in 1 (4%) case, respectively. In 7 (27%) the proof of the infectious agent could not be provided. There was no significant difference in the appearance of the individual bacteria in each group (Figure 3).

## 4. Discussion

The aim of the present study was to investigate the prevalence of oral cavity infections in patients with spondylodiscitis. The incidence of pyogenic spinal infection has increased in recent years. For example, the cause of infection may be an avascular disc, and the infection may then spread progressively through the vertebral body to the subligamentous paravertebral area, epidural space and adjacent vertebral bodies. Diagnosis can be confusing because spondylodiscitis can rarely affect only one vertebral body, with or without disc involvement. Such a diagnosis can have a significant impact on quality of life. In addition to the spinal focus, infections of other organ systems, such as the heart, brain infections and other soft tissues, are more commonly reported.

**Figure 4:**
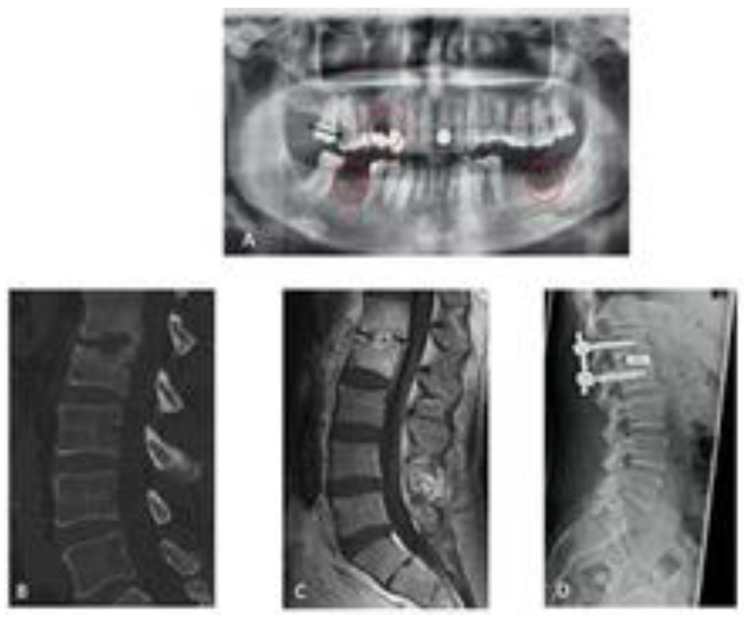
Spondylodisciits and oral cavitiy infection ((**A**) OPG with an infection of the oral cavity/red circels), (**B**,**C**) CT/MRT with bony destruction, (**D**) CT after surgical treatment of discitis).

In addition to treating the spinal infection, optimal care also includes identifying the source of the pyogenic spinal infection as well as the presence of other infections. In a prospectively conducted study with systematic radiological and dental examinations, we were able to demonstrate that more than 50% of the patients with spondylodiscitis undergoing OPG had an infection of the oral cavity (Figure 4).

To the best of our knowledge, the routine use of OPG in the diagnostic workup of patients with spondylodiscitis has thus far not been described. With this study, we were able to show how important it is to assess the dental status of these patients as well. This may be an important step in the detection of a possible source of spinal infections.

The hypothesis that spondylodiscitis is the result of a systemic infection with haematogenous spread of another origin is becoming increasingly important. This hypothesis is supported by the fact that co-infections, such as endocarditis, sepsis or cerebritis, are often found in addition to spondylodiscitis [17,18,19,20]. Consequently, the management of spondylodiscitis should be part of a multidisciplinary approach due to the increasing incidence and new evidence suggesting different foci of haematogenous spread of pathogens [2,21,22].

An important pillar of this approach could also be the treatment of any oral infections that may be present as a likely cause of spinal infection and infective endocarditis. We know from published data that the cause of a spinal infection remains undetected in about 50% of cases [8]. In many of these cases, an odontogenic focus could be the cause. In addition to the spinal focus, patients who had an oral cavity coinfection revealed more often endocarditis, spinal epidural abscesses, sepsis, and even brain abscesses. Thus, we can support the theory of hematogenous spread, possibly from the area of the oral cavity. The presence of an OCI in our study had implications for the duration of treatment, initial and subsequent recovery, and presentation of neurologic symptoms. Although the level of significance was not reached, the length of hospital stay tended to be longer in patients with spondylodiscitis and an oral cavity infection compared with patients without coinfection. The intensity of the pain and neurological status, as well as the length of hospital stay are explained by the fact of the severity of the spinal infection, highlighted by the frequent occurrence of SEA and endocarditis, which in turn may increase the need for further interventions and observations.

Surprisingly, we saw significantly more bony destruction as a consequence of spinal infection in patients without coinfection of the oral cavity. However, further analysis also showed that, in the same group, the time from symptom onset to diagnosis of spinal infection was somewhat longer. Thus, more bony destruction could occur as a result of delayed treatment.

CRP was the only parameter elevated in patients with an OCI. In order to exclude possible confounders, we collected further parameters, in addition to the renal and liver function in the form of creatinine and INR, of the hematopoietic system. We were able to show that the elevation of CRP is indeed a specific parameter of the underlying coinfection. Finally, the examinations of the oral cavity had an enormous clinical relevance as, in more than 40% of cases, the present tooth infection could only be treated by tooth extraction. This work should also be a first approach to determine the necessity and importance of confirming the diagnosis of oral cavity infection in patients with spinal infections. Early co-treatment of the oral cavity could also have important implications for overall treatment duration, recurrent infection, antibiotic load and outcome.

### Limitations

A major limitation of this study is the fact that we did not examine all patients with an OPG to detect OCI. The reasons for this are more practical and include the impossibility of transfer for radiological examination due to severe pain, hemodynamic instability and patient refusal. Furthermore, the ability to stand or sit is a prerequisite for the performance of the OPG. Patients with a neurological deficit could not undergo this examination. Therefore, not all patients could be examined. Additionally, the existence of a selection bias cannot be completely ruled out. Nevertheless, we were able to show in this study that patients with an OCI have a pronounced infection of the spine, increased co-infections of the heart, and also a pronounced pain intensity and neurological deficits. It can therefore be assumed that the number of patients with an OCI could probably be significantly higher than lower. In fact, this assertion remains an assumption. The aim for the future should be to have all patients undergo this or similar screening methods to determine the true prevalence of OCI.

## 5. Conclusions

Based on the results of the present study, we were able to show that the prevalence of oral cavity infections in patients with spinal infections is significant. Thus, oral infections may be an important source of spinal infections and the development of spondylodiscitis, and are associated with morbidity, increased pain, and endocarditis.

## Figures and Tables

**Table 1:** Baseline characteristics of patients with spinal infection and radiological and clinical examination of the oral cavity, *n* = 55.

Patients’ Characteristics	Oral Cavity Infection	No Oral Cavity Infection	*p* Value
*No*, *n (%)*	29	26	
*Age*	63	66	0.9
*Sex*, *male*, *n (%)*	23/79%	18/69%	0.3
***Location***			
*Cervical, n (%)*	5/17%	4/15%	1.0
*Thoracic, n (%)*	6/21%	10/38%	0.2
*lumbar, n (%)*	18/62%	12/46%	0.3
***Affected level***			
*1, n (%)*	18/62%	25/96%	**0.003**
*2, n (%)*	5/17%	1/4%	
*>2, n (%)*	6/21%	0	
*Spinal epidural abscess*, *n (%)*	21/72%	11/42%	0.03
*Bony destruction*, *n (%)*	14/48%	20/77%	0.05
*Positive blood culture*, *n (%)*	15/52%	8/31%	0.2
*Endocarditis*, *n (%)*	9/31%	1/4%	**0.01**
*Cardiac surgery*, *n (%)*	5/59%	0	1.0
*Diabetes*, *n (%)*	6/21%	2/8%	0.3
*Sepsis*, *n (%)*	3/10%	0	0.2
*HIV*, *n (%)*	1/3%	0	1.0
*Obesity*, *n (%)*	9/31%	7/27%	1.0
*IDU*, *n (%)*	2/7%	1/4%	1.0
*CCI*, *median*	3.5	3	0.4
*ASA*, *median*	3	3	0.3
*BMI*, *median*, *kg/cm^2^*	27	29	0.7
*Median time until diagnosis*, *weeks*	1	2	0.8
*LOH*, *median in days*	22.5	16.5	0.2
*Brain abscess*	1/3%	0	1.0
*CRP*, *median*	15	8.4	0.09
*WBC*, *median*	9	10	0.7
*Hemoglobin*, *median*	10.8	11.9	0.4
*Creatinine*, *median*	0.7	0.8	0.7
*Thrombocyte*, *median*	303	282	0.9
*INR*, *median*, *%*	1.2	1.1	0.8
*Bacteria detection possible*	23/79%	19/73%	0.8
***VAS, median***			
*Admission*	9	8	**0.02**
*Postoperatively*	5	5	0.08
*3 months*	1	0	**0.02**
*In hospital mortality*, *n (%)*	0	0	
***Functional status at admission***			
*ASIA A*	4/14%	1/4%	
*ASIA B*	0	0	
*ASIA C*	3/10%	1/4%	
*ASIA D*	5/17%	0	
*ASIA E*	17/59%	24/92%	**0.005**
*surgery*	20/69%	20/77%	0.5
*Staphylococcus*	10/34%	13/50%	0.2
*Streptococcus*	6/21%	5/19%	1.0
*Enterococcus*	3/3%	1/4%	0.6
*Misc.*	4/14%	0/27%	0.1
*No proof*	6/21%	7/27%	0.8

## Data Availability

The raw data supporting the conclusions of this article will be made available by the authors, without undue reservation.
